# Electronic Cigarette Prevalence and Knowledge Among Medical Students in Saudi Arabia and Bahrain: A Cross-National Study

**DOI:** 10.7759/cureus.45583

**Published:** 2023-09-20

**Authors:** Yosra A Turkistani, Maryam Dahlawi, Rahaf I Bukhari, Muhammad Aldabbagh, Yasir A Turkistani, Arwa Malosh

**Affiliations:** 1 Department of Medicine, College of Medicine, Umm Al-Qura University, Makkah, SAU; 2 Faculty of Medicine, Umm Al-Qura University, Makkah, SAU; 3 Hematology/Oncology, King Salman Bin Abdulaziz Medical City, Madinah, SAU; 4 Family Medicine, Al-Badrani Primary Health Care Center, Madinah, SAU

**Keywords:** medical students, alternative tobacco products, smoking, vapor devices, electronic cigarettes

## Abstract

Background

Electronic cigarettes (e-cigarettes and vapour devices) are the most commonly used form of alternative tobacco products (ATPs). The use of these vapour devices has been dramatically increasing worldwide, especially among current and former traditional cigarette smokers. A strong influence that will affect patients’ health attitudes and play a crucial role in tobacco control and smoking cessation are medical students, as they are future physicians. Therefore, in our study, we aimed to determine the prevalence rate of e-cigarettes and the level of knowledge among medical students in Saudi Arabia and Bahrain.

Methods

We conducted a cross-sectional study of medical students in Saudi Arabia and Bahrain using an online survey. This was distributed through social media platforms such as Twitter, WhatsApp, Telegram, and Facebook. Medical students of all years were included. The questionnaire was adapted from two previous studies.

Results

The study enrolled 1730 medical students. The majority of the participants did not recommend the use of e-cigarettes as a method for smoking cessation or believed that these types of ATPs lower the risk of cancer in comparison with traditional cigarettes. The data showed a strong association between gender and e-cigarette smoking, with the majority of current smokers being men (n = 184) and experimental smokers being women (n = 800). Moreover, an educational gap was discovered, as few of the students had received an education in their medical school’s curriculum on the use of e-cigarettes. There was a significant association between receiving an education at a medical school and having adequate knowledge of e-cigarettes.

Conclusion

The increasing number of e-cigarette users among medical students is concerning. Our study showed that students are not receiving a decent education on the use of ATPs during medical school, which urges further adjustment of the curriculum. This will play a huge role in their behaviour and the provision of future treatment plans to patients as physicians.

## Introduction

Smoking tobacco has been a significant and persistent global social issue. According to the World Health Organisation, tobacco consumption leads to more than eight million deaths each year, impacting approximately half of its users. Out of these, direct tobacco use is responsible for over seven million deaths, while around 1.2 million deaths are attributed to non-smokers being exposed to second-hand smoke. Tobacco smoking is a well-established risk factor for various health conditions, including cardiovascular diseases, lung disorders, cancer, diabetes, and hypertension, leading to premature death [1]. Tobacco use is the leading preventable cause of death in the United States and globally.

Apart from traditional cigarettes, a variety of alternative tobacco products (ATPs) are now available. These include hookahs and waterpipes, in which tobacco mixed with molasses is smouldering in the bowel of the hookah and then drawn through water and inhaled; chewing tobacco and snus, which are smokeless tobacco products that come in loose-leaf strips of shredded tobacco leaves; battery-powered electronic cigarettes (e-cigarettes and vapour devices) that deliver nicotine through a cartridge and enable the user to exhale nicotine vapour from the device's end; kreteks, which are clove cigarettes that contain 60-90% tobacco; and cigars, which are large bundles of tobacco tightly rolled in leaf tobacco and then smoked. Previous research has indicated that specific ATPs, such as hookahs, deliver higher levels of tar, nicotine, and carbon monoxide than cigarettes and are likely associated with adverse health effects comparable to or more severe than those of cigarettes [2].

E-cigarettes are the most commonly used ATPs, and there has been a dramatic increase in their use worldwide. The number of adults reporting current use of e-cigarettes has more than doubled. The use of e-cigarettes is more common among current and former smokers than never-smokers, especially as they have been introduced into the market in recent years [3-5]. However, their sale is forbidden to people under the age of 18, and advertising electronic devices is prohibited, as is their use in certain places such as schools and closed forms of public transport [6].

Physicians have a vital role in screening, preventing, and supporting patients in quitting smoking, as well as promoting a healthy lifestyle. They possess the capacity to influence patient behaviour and carry the responsibility to contribute towards further reducing tobacco usage in general. Studies have shown that physicians who personally use tobacco are less likely to provide cessation counselling to patients [2,7]. Medical students are future physicians who will influence the health attitudes of patients and play a crucial role in tobacco control decision-making at various levels, from community to nation, by offering a cessation counselling option. Therefore, their use of, knowledge of, and beliefs about tobacco and ATPs are of vital importance because their willingness to provide prevention counselling is heavily influenced by their own habits, knowledge, and beliefs [2,8]. Hence, designing a medical school curriculum on cigarettes and ATPs is necessary, as there is little information on medical students’ knowledge of and attitudes towards e-cigarettes, and their education on tobacco product use is unsatisfactory. This particularly concerns students because their habits and health behaviours are formed during their studies [9-12].

Data on e-cigarette smoking use among Saudi Arabian medical students is lacking. Because of the sharp increase in e-cigarette use and as medical students are future healthcare providers who will play a critical role in health promotion and smoking cessation for the general population, this study aims to determine the prevalence rate of e-cigarettes, level of knowledge, and reasons for experimenting with e-cigarettes among medical students in Saudi Arabia and Bahrain.

## Materials and methods

Study design and participants

This cross-sectional study used an online survey conducted in Saudi Arabia and Bahrain from July to November 2022. The study enrolled medical students attending medical schools in Saudi Arabia and Bahrain. The study was conducted by the Faculty of Medicine at Umm Al-Qura University, Makkah, Saudi Arabia.

Sampling strategy

According to the Raosoft sample size calculation, the estimated sample size to reach a precision of 5% with a 95% confidence interval was 385 participants. Our study included medical students ranging from their first year to their internship year, representing various medical schools in all regions of Saudi Arabia and Bahrain. However, medical students who were neither residents nor citizens of Saudi Arabia or Bahrain were not included in the study. We provided an online link for the survey published on social media platforms such as WhatsApp, Twitter, Telegram, and Facebook. Additionally, a clear explanation of the inclusion criteria was given at the beginning of the survey, where respondents were asked if they were medical students and about their country of residence. Those who provided answers outside our predefined criteria were excluded from the study. No identifier information was asked. Only research staff were authorised to access the data, ensuring participants’ privacy and confidentiality.

Study tool

The study enrolled 1730 participants. The web-based self-reported questionnaire was adapted from two previously published studies [13,14]. It was composed of four parts, including (i) consent to participate in the study, (ii) demographic data, (iii) knowledge and attitudes of medical students towards electronic cigarettes as well as their education, and (iv) their perception of the use of electronic cigarettes. After structuring the questionnaire using Google Forms, data were collected for 1 month, from November 12 to December 12, 2022. They were kept safe with authorised access only.

Statistical analysis

The obtained data were initially gathered in an Excel sheet to be checked. Afterward, we used IBM SPSS Statistics for Windows, Version 23.0 (released 2015; IBM Corp., Armonk, New York, United States) for the data analysis. The categorical data were presented as frequencies and percentages, and the numerical data as means and standard deviations. The Chi-square test was used to compare the categorical variables, and a P-value of ≤ 0.05 was considered to be statistically significant.

Ethical consideration

The Biomedical Ethics Committee at the College of Medicine of Umm Al-Qura University, Makkah, Saudi Arabia, granted approval for this study on November 11 (IRB: HAPO-02-K-012-2022-22-1242). Prior to commencing the questionnaire, each participant provided electronic informed consent.

## Results

Of the 1730 participants, the majority (71.3%) were aged between 18 and 25 years. More than half of the study sample were women (60.1%). Altogether, (27.7%) of the students were in their internship year, followed by (15.3%) and (14%) who were 4th-year and 5th-year students, respectively. Moreover, 32.1 percent of the study population was from Bahrain, while 67.9% were from Saudi Arabia. Most of the medical students had not received any education about e-cigarettes at medical school (64.1%), while the rest (35.9%) stated they had received some education. The details of the study characteristics are listed in Table 1.

**Table 1:** Demographics characteristics of medical students SA: Saudi Arabia

Variables	Frequency	Percentage
Age		
<18 years	126	7.3%
18-25 years	1234	71.3%
>25 years	370	21.4%
Gender		
Male	690	39.9%
Female	1040	60.1%
Academic year in medical school		
Year 1/preparatory year	140	8.0%
Year 2	154	8.9%
Year 3	238	13.8%
Year 4	265	15.3%
Year 5	242	14.0%
Year 6	212	12.3%
Intern	479	27.7%
Region of residency		
Eastern region of SA	289	16.6%
Central region of SA	152	8.8%
Northern region of SA	72	4.2%
Western region of SA	283	16.4%
Southern region of SA	379	21.9%
Bahrain	555	32.1%
Receiving any education about electronic cigarettes in medical school		
Yes	621	35.90%
No	1109	64.1%

The medical students included in our study were asked questions to assess their knowledge of and attitudes towards the use of e-cigarettes. As shown in Table 2, 54.1 percent of the participants did not recommend the use of e-cigarettes for patients as a smoking cessation method. By comparison, 18.2% did recommend this approach for patients. A total of 23.9 percent of the medical students believed that e-cigarettes were a helpful aid for smoking cessation, while 52.9 percent did not. In addition, 22.9 percent of the students believed that the use of e-cigarettes lowers the risk of cancer in patients who use them instead of smoking traditional cigarettes. On the other hand, most of the students (49.7%) did not agree with this statement. One-third of the medical students neither agreed nor disagreed about being confident in their ability to discuss e-cigarette use with their patients, and 38.1% were confident enough in their ability to discuss traditional cigarette use with their patients. Most of the respondents (46.5%) strongly agreed with the importance of physicians being educated about e-cigarettes. In total, 38.6% of the medical students agreed that e-cigarettes are addictive, 29.4% strongly agreed, and 20.3% disagreed. With regard to the use of e-cigarettes being better for patients than traditional smoking, 36.7% are neither agreed nor disagreed, and 19.3% agree.

**Table 2:** Knowledge and attitude of medical students regarding electronic cigarettes

Variables	Frequency	Percentage
If you were talking to a patient who smokes cigarettes today, would you recommend the use of e-cigarettes as a smoking cessation method?		
Yes	316	18.2%
No	935	54.1%
Not sure	479	27.7%
Do you believe e-cigarettes are a helpful aid of smoking cessation?		
Yes	414	23.9%
No	915	52.9%
Not sure	401	23.2%
Do you believe that e-cigarettes lower the risk of cancer for patients who use them instead of smoking traditional cigarettes?		
Yes	396	22.9%
No	860	49.7%
Not sure	474	27.4%
As a student, I feel confident about my ability to discuss e-cigarettes use with my patients		
Strongly disagree	169	9.8%
Disagree	265	15.3%
Neither agree or disagree	570	33%
Agree	466	26.9%
Strongly agree	260	15.0%
As a student, I feel confident about my ability to discuss traditional cigarettes use with my patients		
Strongly disagree	180	10.4%
Disagree	181	10.5%
Neither agree or disagree	350	20.2%
Agree	659	38.1%
Strongly agree	360	20.8%
It is important for physicians to be educated about e-cigarettes		
Strongly disagree	108	6.2%
Disagree	82	4.7%
Neither agree or disagree	216	12.5%
Agree	521	30.1%
Strongly agree	803	46.5%
E-cigarettes are addictive		
Strongly disagree	89	5.1%
Disagree	114	6.6%
Neither agree or disagree	352	20.3%
Agree	668	38.6%
Strongly agree	507	29.4%
Despite the unknowns, the use of electronic cigarettes is better for my patients than traditional smoking		
Strongly disagree	326	18.8%
Disagree	287	16.6%
Neither agree or disagree	635	36.7%
Agree	334	19.3%
Strongly agree	148	8.6%

In regard to the association between e-cigarette smoking and the demographic characteristics of the respondents (Table 3), there was a significant association between gender and e-cigarette smoking status (p<0.001). As the majority of current smokers were men (n = 184), women were more experimental smokers, which is defined as a temporary or occasional engagement in smoking behaviour for the purpose of exploration, curiosity, or social influence. It involves individuals who may not consider themselves regular smokers but may try smoking cigarettes or other tobacco products on an experimental basis. Experimental smoking is distinct from regular or habitual smoking, as it does not typically involve a long-term commitment to smoking and may not develop into a consistent or addictive pattern of tobacco use. Furthermore, the majority of the respondents who had never tried e-cigarettes were women (n = 800). Moreover, on the association between e-cigarette smoking habits and academic year at medical school, most of the current smokers were in the 6th academic year (n = 55), while a higher number of experimental smokers were in the 5th academic year (n = 35). All these associations were significant (p <0.001). In regard to the region of residence, most of the respondents who were current smokers were from the southern region of Saudi Arabia (n = 67), most of the experimental smokers were from the western region of Saudi Arabia, and most of the respondents who had never tried e-cigarettes were from Bahrain (p<0.001). When comparing e-cigarettes with traditional cigarette smoking, most of the respondents who were current smokers of e-cigarettes were current traditional cigarette smokers as well (n = 90). Furthermore, (n = 62) respondents who were current e-cigarette smokers were former smokers of traditional cigarettes, while (n = 65) respondents were experimental smokers of e-cigarettes and traditional cigarettes together. The majority of the respondents had never tried either electronic or traditional cigarettes (n = 1088, p<0.001).

**Table 3:** Relationship between smoking electronic cigarettes and demographics characteristics SA: Saudi Arabia

Demographics characteristics of medical students	Electronic cigarettes smoking				
	Current smoker	Former smoker	Experimental smoker	Never tried smoking	P-value
Age					
<18 years	32	16	8	70	P=.001
18-25 years	175	71	126	862	
>25 years	74	33	26	237	
Gender					
Male	184	65	72	369	P=.001
Female	97	55	88	800	
Academic year in medical school					
Year 1	29	14	8	89	P=.001
Year 2	22	12	7	113	
Year 3	34	9	25	170	
Year 4	36	21	32	176	
Year 5	53	22	35	132	
Year 6	55	17	25	115	
Intern	52	25	28	374	
Region of residency					
Eastern region of SA	59	17	18	195	P=.001
Central region of SA	34	15	24	79	
Northern region of SA	21	16	12	23	
Western region of SA	49	18	37	179	
Southern region of SA	67	25	35	252	
Bahrain	51	29	34	441	
Traditional cigarettes smoking					
Current smoker	90	25	16	20	P=.001
Former smoker	62	36	23	23	
Experimental smoker	74	39	65	38	
Never tried smoking	54	20	57	1088	

Table 4 presents the association between receiving an education on e-cigarettes at medical school and medical students’ knowledge, attitudes, and smoking habits. Those students who had received an education at medical school would not recommend the use of e-cigarettes as a smoking cessation method for their patients (n = 365), while the majority of the respondents who had not received an education were unsure about recommending the use of e-cigarettes as a smoking cessation method (n = 935, p<0.001). Most of the respondents who had both received and not received an education did not believe that e-cigarettes are a helpful aid for smoking cessation or that e-cigarettes lower the risk of cancer for patients who use them instead of smoking traditional cigarettes (n = 310, 605, and n = 271, 589, respectively, p<0.001). In regard to the medical students’ confidence in their ability to discuss e-cigarette use with their patients, most of those who had received an education were confident (n = 205), while most uneducated respondents neither agreed nor disagreed (n = 439, p<.001). The majority of these educated and uneducated respondents agreed that they feel confident in their ability to discuss traditional cigarette use with their patients (n = 218, 441, p =0.004). Moreover, most strongly agreed that it is important for physicians to be educated about e-cigarettes (n = 270, 533, p = 0.052). The educated students strongly agreed that e-cigarettes are addictive (n = 238), as did the uneducated ones (n = 476, p<0.001). Regarding the medical students’ agreement with e-cigarettes being better for their patients than traditional smoking, the majority of the educated ones disagreed with this statement (n = 181), while most of the uneducated respondents neither agreed nor disagreed (n = 482, p<0.001).

**Table 4:** Relationship between teaching medical students about electronic cigarettes and their level of knowledge, attitude, and smoking habits

Variables		Receiving any education about electronic cigarettes in medical school		
Medical students knowledge and attitude:		Yes	No	P-value
If you were talking to a patient who smokes cigarettes today, would you recommend the use of e-cigarettes as a smoking cessation method?				P=0.001
Yes		97	219	
No		365	470	
Not sure		159	935	
Do you believe e-cigarettes are a helpful aid of smoking cessation?				P=0.001
Yes		191	223	
No		310	605	
Not sure		120	281	
Do you believe that e-cigarettes lower the risk of cancer for patients who use them instead of smoking traditional cigarettes?				P=0.001
Yes		188	208	
No		271	589	
Not sure		162	313	
As a student, I feel confident about my ability to discuss e-cigarettes use with my patient				P=0.001
Strongly disagree		58	111	
Disagree		73	192	
Neither agree or disagree		131	439	
Agree		205	261	
Strongly agree		154	106	
As a student, I feel confident about my ability to discuss traditional cigarettes use with my patients				P=0.004
Strongly disagree		72	108	
Disagree		68	113	
Neither agree or disagree		105	245	
Agree		218	441	
Strongly agree		158	202	
It is important for physicians to be educated about e-cigarettes				P=0.052
Strongly disagree		51	57	
Disagree		39	43	
Neither agree or disagree		79	137	
Agree		182	339	
Strongly agree		270	533	
E-cigarettes are addictive				P=0.001
Strongly disagree		48	41	
Disagree		39	75	
Neither agree or disagree		104	248	
Agree		192	476	
Strongly agree		238	269	
Despite the unknowns, the use of electronic cigarettes is better for my patients than traditional smoking				P=0.001
Strongly disagree		181	145	
Disagree		104	183	
Neither agree or disagree		153	482	
Agree		110	224	
Strongly agree		73	75	
Smoking status:				
Electronic cigarettes smoking				P=0.001
Current smoker		142	139	
Former smoker		68	52	
Experimental smoker		74	86	
Never tried smoking		437	732	
Traditional cigarettes smoking				P=0.001
Current smoker		77	74	
Former smoker		68	76	
Experimental smoker		111	105	
Never tried smoking		365	854	

**Figure 1:**
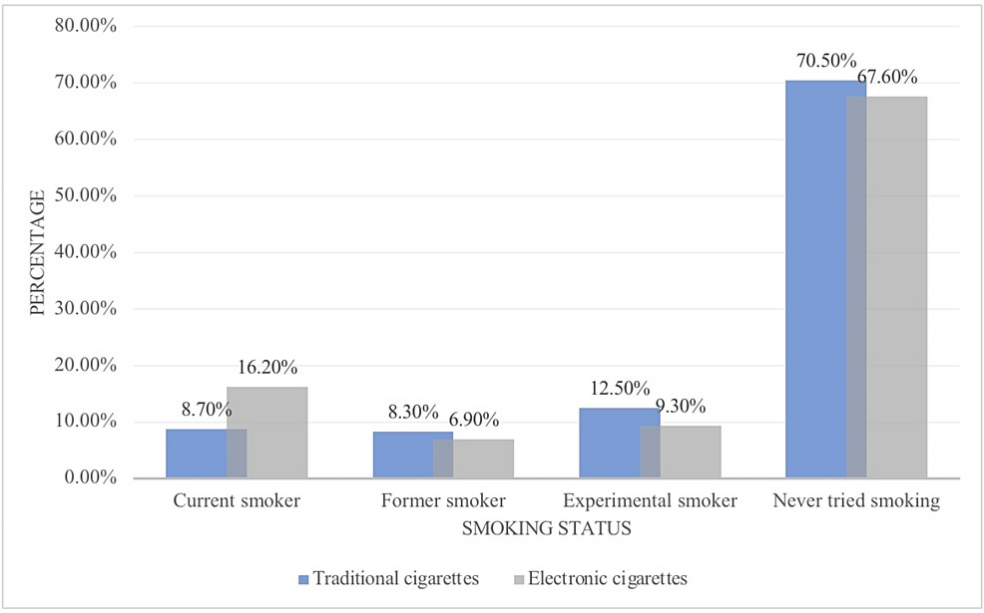
Prevalence of cigarettes smoking

**Figure 2:**
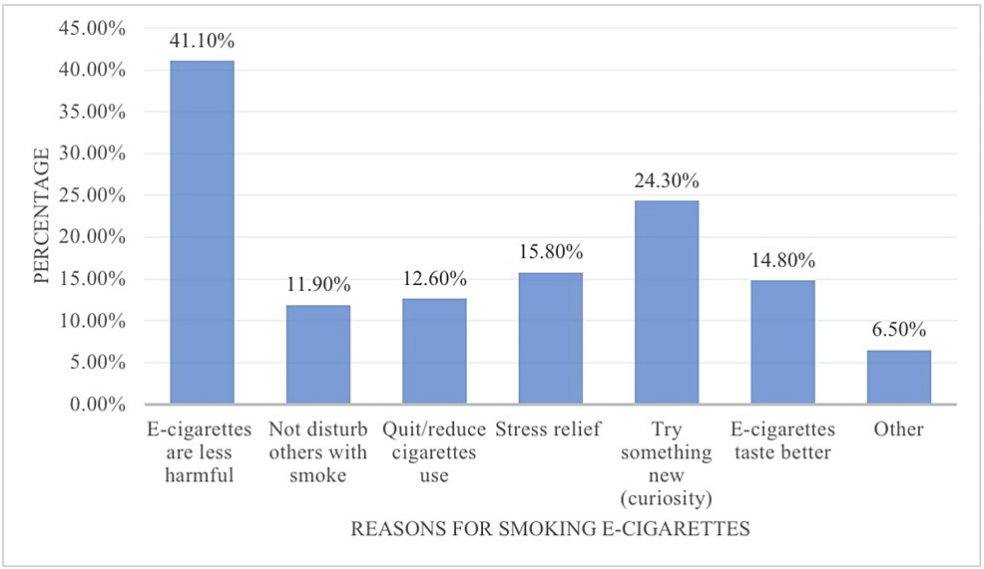
Reasons why medical students smoke electronic cigarettes

Figure 1 presents the prevalence rate of smoking e-cigarettes among medical students. As shown, 67.6% had never tried e-cigarettes, 9.3% had tried them, and 6.9% were former smokers. Furthermore, the prevalence rates of traditional cigarette smoking among the medical students were as follows: most had never tried traditional smoking (64.7%), some were experimental smokers (12.5%), and only 8.3% were former smokers. Of the medical students who were current smokers, only 8.7% smoke traditional cigarettes, while twice as many (16.2%) smoke e-cigarettes. The most frequently reported reasons for smoking e-cigarettes are shown in Figure 2. Many of the students smoke e-cigarettes because they think they are less harmful (41.1%), and 24.3% have smoked e-cigarettes to try something new (curiosity). Fewer of the respondents have smoked e-cigarettes to relieve stress, as they taste better, to quit/reduce traditional cigarette use, and to not disturb others with smoke, with proportions of 15.8%, 14.8%, 12.6%, and 11.9%, respectively.

## Discussion

The use of e-cigarettes has increased dramatically recently, and little is known about their use by healthcare providers; thus, this study aimed to determine the prevalence rate of e-cigarettes, level of knowledge, and reasons for experimenting with e-cigarettes among medical students in Saudi Arabia and Bahrain. This research’s results reported that out of the 1730 participants, most were aged 18-25 years (71.3%), 60.1% were women, and 27.7% were interns. These demographic characteristics are similar to those of a study conducted at an urban U.S. university among nursing students, most of whom were women (88.4%) [7]. Similarly, another study of medical students in the U.S. [14] reported that most of their participants were women (58%).

In this study, we examined the prevalence rate of e-cigarettes. Out of all medical students surveyed, 32.4% reported using e-cigarettes; among these e-cigarette users, 16.2% were current smokers, 9.3% had tried smoking e-cigarettes, and 6.9% were former e-cigarette smokers. Compared with the prevalence rate of traditional cigarettes among the medical students in our study, of the total respondents, 29.5 percent reported smoking traditional cigarettes. Within this group, 12.5% had tried smoking, 8.7% were current smokers, and 8.3% were former smokers. These results show that the prevalence rate of e-cigarettes is higher than that of traditional smoking among these medical students. In the U.S., the rate of cigarette smoking has declined dramatically over the past several decades. However, the Centres for Disease Control and Prevention (CDC) issued a recent warning about the increasing use of ATPs, including e-cigarettes, which have become the preferred method of tobacco and nicotine delivery among teenagers. The rise in e-cigarette use among adolescents has been considerably higher than the already low rate of traditional tobacco use. Moreover, a study conducted among U.S. students showed that (40-54%) have used ATPs [15], strongly consistent with our findings.

The dramatic increase in the prevalence rate needs to be explored more. Thus, we asked the medical students about why they smoke e-cigarettes. The majority smoke e-cigarettes because they think that they are less harmful (41.1%), (24.3%) smoke because they want to try something new (curiosity), (15.8%) smoke to relieve stress, (14.8%) say that e-cigarettes taste better, and (12.6%) use e-cigarettes to reduce and quit traditional cigarette smoking. This agrees with a study conducted among Montana adults in collaboration with the CDC [9], which showed that the most common reasons are to try something new (curiosity) (64%), to quit or reduce cigarette use (56%), and because e-cigarettes are less harmful than traditional cigarettes (52%).

E-cigarette users fall into two main categories: those who are smokers trying to quit traditional cigarettes and those who use e-cigarettes for recreational purposes. The latter group considers e-cigarettes a recreational tool for several reasons. Factors such as the availability of different flavours, advertising, and lower perceived risks are believed to be significant factors that encourage the use of e-cigarettes for recreational purposes. Artificial flavours can be created through chemical synthesis to imitate desirable aromas or directly extracted from natural components. Therefore, the properties of these flavours determine their specific level of toxicity to humans. However, the use of e-cigarettes has been linked to toxic damage that can cause bronchiolitis obliterans. Moreover, the latest generation of e-cigarette products has notably higher levels of serum nicotine than previous generations. Additionally, these products are highly accessible on the market and easy to use on a daily basis, resulting in higher concentrations of inhaled nicotine. This increasing popularity of e-cigarettes has corresponded with a rise in cases of lung injury associated with vaping. This has brought national attention to the potential respiratory harm of e-cigarette use and has created confusion about their safety. [16]. All this refutes the idea that e-cigarettes are harmless. Indeed, they are considered to be more harmful than traditional cigarettes, as there is a large amount of unfamiliarity with newer products and a lack of prevention education.

This study explored the association between smoking e-cigarettes and the demographic characteristics of our participants. We found a gender difference, as those who use e-cigarettes are predominantly men. The prevalence rate of e-cigarettes is higher among older medical students (p<.001). This poses a significant risk as older students serve as role models for younger students, who may imitate their behaviour and follow their habits. Regarding the association between traditional and e-cigarette smoking, a high number of current e-cigarette smokers are concurrent traditional cigarette smokers or former traditional smokers. A recent study [17] suggested that former traditional smokers who continue to use e-cigarettes are more likely to resume smoking traditional cigarettes. In addition, using e-cigarettes without the intention of quitting smoking may lead to dual use.

More than half of our medical students have not received any medical education on e-cigarettes at medical school (64.1%). This lack of education has impacted their knowledge, attitudes, and confidence in addressing and discussing e-cigarette use with patients. (p<.001). Therefore, there is an educational gap in medical schools. By contrast, participants are being exposed to information about e-cigarettes outside of the medical school curriculum, emphasising the need to provide accurate and comprehensive information to students during their medical education. As future carers, medical students will play an important role in health promotion and disease prevention. Hence, designing a medical school curriculum on cigarettes and ATPs is necessary to be added to medical school curricula [14].

Previous studies indicate that healthcare providers who smoke are less likely to evaluate and advise their patients on smoking cessation. This is because a provider's willingness to provide prevention counseling is strongly influenced by their personal habits, knowledge, and beliefs [18]. This particularly concerns students because habits and health behaviours are formed during medical school [2]. Providing education to healthcare providers on e-cigarettes can enhance their knowledge, attitudes, confidence, cessation counselling skills, and screening abilities. This, in turn, has the potential to influence patient behaviour and increase their responsibility to promote the reduction of tobacco product use. The importance of healthcare providers' knowledge in this matter cannot be overstated [19]. A survey involving primary care physicians in the U.S. found that those who recommend the use of e-cigarettes for smoking cessation are more likely to be men and have higher confidence in their e-cigarette counselling skills, suggesting that confidence and gender could play a role in physicians’ behaviours regarding e-cigarettes [14,20]. Thus, we recommend further studies to investigate the factors that influence medical students’ confidence, attitudes, and behaviour regarding e-cigarettes. Expanding studies of the younger population is another recommendation.

It is crucial to recognise and address the limitations inherent in this study. Firstly, the potential for selection bias exists due to the reliance on voluntary participation, which may introduce a bias in the representation of medical students across the two countries. Consequently, the findings may not be generalizable to the entire population of medical students in Saudi Arabia and Bahrain. Secondly, the use of self-reported data collected through an online survey introduces the possibility of response bias. Participants may provide inaccurate or biased information, consciously or unconsciously. These limitations should be carefully considered when interpreting and drawing conclusions from the study's results, and further research with more diverse and representative samples is recommended to enhance the robustness of the findings.

## Conclusions

A concerning proportion of medical students were found to use e-cigarettes in our study, and we identified a gap in their medical education on the topic. The majority of medical students have not received any formal education on their curriculum regarding e-cigarettes, which has resulted in inadequate knowledge, attitudes, confidence, and skills necessary for providing screening, prevention, and cessation counselling to patients. Given the expected rise in e-cigarette use, it is crucial to develop a medical school curriculum that includes education on cigarettes and alternative tobacco products.
